# Results of 189 wrist replacements: A report from the Norwegian Arthroplasty Register

**DOI:** 10.3109/17453674.2011.588858

**Published:** 2011-09-02

**Authors:** Yngvar Krukhaug, Stein A Lie, Leif I Havelin, Ove Furnes, Leiv M Hove

**Affiliations:** ^1^The Norwegian Arthroplasty Register, Department of Orthopaedic Surgery, Haukeland University Hospital; ^2^Section for Orthopaedic Surgery, Department of Surgical Sciences, University of Bergen; ^3^Department of Health, University Research Bergen, Bergen, Norway; Correspondence: yngvar.krukhaug@helse-bergen.no

## Abstract

**Background and purpose:**

There is very little literature on the long-term outcome of wrist replacements. The Norwegian Arthroplasty Register has registered wrist replacements since 1994. We report on the total wrist replacements and their revision rates over a 16-year period.

**Material and methods:**

189 patients with 189 primary wrist replacements (90 Biax prostheses (80 of which were cementless), 23 cementless Elos prostheses, and 76 cementless Gibbon prostheses), operated during the period 1994–2009 were identified in the Norwegian Arthroplasty Register. Prosthesis survival was analyzed using Cox regression analyses. The 3 implant designs were compared and time trends were analyzed.

**Results:**

The 5-year survival was 78% (95% CI: 70–85) and the 10-year survival was 71% (CI: 59–80). Prosthesis survival was 85% (CI: 78–93) at 5 years for the Biax prosthesis, 77% (CI: 30–90) at 4 years for the Gibbon prosthesis, and 57% (CI: 33–81) at 5 years for the Elos prosthesis. There was no statistically significant influence of age, diagnosis, or year of operation on the risk of revision, but females had a higher revision rate than males (RR = 3, CI: 1–7). The number of wrist replacements performed due to osteoarthritis increased with time, but no such change was apparent for inflammatory arthritis.

**Interpretation:**

The survival of the total wrist arthroplasties studied was similar to that in other studies of wrist arthroplasties, but it was still not as good as that for most total knee and hip arthroplasties. However, a failed wrist arthroplasty still leaves the option of a well-functioning arthrodesis.

The body of literature on wrist arthroplasty is increasing, but most studies have dealt with a single prosthesis design or have compared the outcome of wrist prosthesis to that of wrist fusion. Furthermore, most studies have been on rheumatoid patients. To our knowledge, no randomized trials comparing different designs have ever been published.

Wrist arthroplasty with silicone implants was first popularized by Swanson in the 1960s (Swanson 1973). The early results of these were promising. Unfortunately, with longer follow-up, mechanical failure became apparent and severe inflammatory reaction caused by silicone disintegration ensued ( Smith et al. 1985, Brase and Millender 1986, Jolly et al. 1992).

The second-generation wrist prostheses introduced in the 1970s typically included two metal components that articulated by means of a ball-and-socket or a hemispheric design (Meuli 1973, Volz 1976). Most of these prostheses were taken off the market because of problems of joint imbalance and dislocation (Lorei et al. 1997, Carlson and Simmons 1998, Vogelin and Nagy 2003).

The third-generation of wrist prostheses represents an effort to reconstruct the center of wrist motion in order to prevent imbalance and dislocation (Cavaliere and Chung 2008).

The results of total wrist arthroplasy in terms of prosthesis survival have generally been poor compared to most other prostheses. In 2 studies in which the Biax prosthesis—a cementless third-generation implant—was used, the 5-year survival was found to be 83% (Cobb and Beckenbaugh 1996), and the 8-year survival was also 83% (Takwale et al. 2002).

In this study, we estimated the incidence, prosthesis survival, and causes of and risk factors for revision of wrist arthroplasties using data from the population-based Norwegian Arthroplasty Register.

## Patients and methods

The Norwegian Arthroplasty Register (NAR) started to collect data on total hip replacements in 1987. In 1994, this register was extended to include all artificial joints (Havelin 1999). Individual reports are received from all 7 hospitals that perform total wrist replacements in the country (population: 4.8 million).

**Figure 1.**
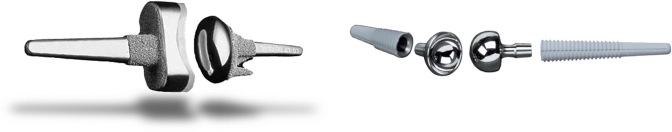
A. The Biax prosthesis. B. The Gibbon prosthesis.

From 1994 through 2009, 189 primary total wrist replacements were performed in 189 patients (Table 1). 3 types of wrist prostheses were used: “Biax”, “Elos”, and “Gibbon” (Figure 1). The Biax prosthesis (DePuy, Warsaw, IN) is a 3-component prosthesis consisting of distal and proximal porous-coated metal parts and a UHMW polyethylene sliding core. The Biax prosthesis was used in the period 1994–2005, and without cement in 80 of 90 cases.

**Table 1.** Demography

A	B	C	D	E	F	G	H	I	J
Biax	90	89%	57 (28–77)	6	84	5	18 (1–46)	18	9.3 (0–11.6)
Elos	23	39%	55 (23–79)	23	0	2	12 (2–21)	10	4.6 (0.4–8.6)
Gibbon	76	58%	52 (17–79)	44	32	3	25 (9–49)	11	2.6 (0.1–4.0)
Total	189	71%	55 (17–79)	73	116	7	27 (1–70)	39	5.4 (0–11.6)

**A** Type of prosthesis **B** No. of primary prostheses **C** Proportion of females **D** Mean age (range) **E** Non-inflammatory group (n) **F** Inflammatory group (n) **G** No. of hospitals **H** Mean operations per hospital (range) **I** No. of revisions **J** Median follow-up in years (range)

3 versions of the Elos prosthesis were used (Elos 1: n = 2; Elos 2: n = 6; and Elos 3: n = 15). The 3 versions were all preliminary types of the Gibbon prosthesis. Elos 1 had a short metacarpal screw which was fully threaded, as was the radial screw. In the later versions of the Elos prosthesis, the metacarpal screws were longer, the diameter smaller, and the threads lower. The Elos implants were used in the period 2000–2005 and without cement in all cases.

The Gibbon prosthesis (Swemac, Linkoping, Sweden) was CE-marked in late 2005, and the design has not changed since. The Gibbon prosthesis changed name to Motec in 2010, without any change to the prosthesis. The Gibbon has a smaller screw diameter, and the threaded area has been changed compared to the Elos 3. The Gibbon prosthesis is a modular (4-component) prosthesis. The articulation is cobalt chrome-molybdenum alloy treated with chromium nitride, and the stem is made of titanium alloy blasted and coated with Bonit—a resorbable calcium phosphate combination (Brush white). Gibbon prostheses were used from 2006 to the end of inclusion, and all were inserted without cement.

The diagnoses were grouped into “inflammatory arthritis” (I) (n = 116) comprising rheumatoid arthritis and psoriatic arthritis, and into “non-inflammatory arthritis” (NI) (n = 73) comprising primary osteoarthritis, post-fracture disorders, ligament injuries, and joint destruction after infection.

### Statistics

The observation time was the time from the primary replacement until revision, or until the end of the study or death. The date of death for the patients who died was obtained from Statistics Norway (www.ssb.no/english/). Median follow-up (observation) time was calculated using the reverse Kaplan-Meier method (Schemper and Smith 1996).

A revision was defined as exchange or removal of the whole prosthesis or parts of the prosthesis.

We used the Student t-test and analysis of variance (ANOVA) to compare continuous variables. For comparison of categorical variables, Chi-square tests were used. All p-values were 2-tailed, and the significance level was set to 0.05. In the Kaplan-Meier survival curves, the endpoint was revision for any reason. The survival curves were presented with log-transformed 95% confidence intervals (CIs) and a lower limit adjustment for the number of patients at risk (Dorey and Korn 1987). The survival curves were ended at 10 years or when 5 cases remained, whichever came first.

Differences in revision rates between groups were tested using the log-rank test. Cox multiple regression analyses were used to study relative risks (RRs; hazard rate ratios) of revision according to prosthesis type, diagnosis, age, and sex. All relative risks were adjusted for the other variables.

Poisson regression analysis was used to analyze trends in the incidence of wrist replacement procedures. These analyses were performed based on annual population rates for the Norwegian population, obtained from Statistics Norway. The p-values given in the text correspond to values derived from these Poisson analyses. Analyses were done using SPSS software version 15 and the program “R”.

## Results

**Figure 2.**
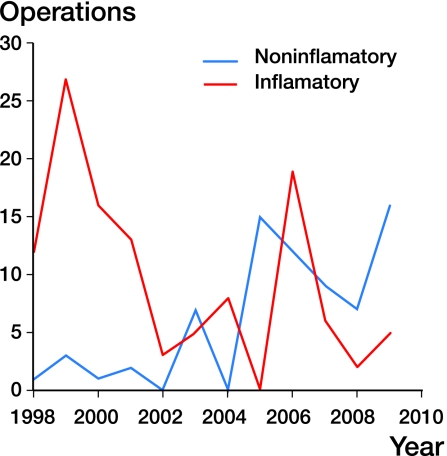
Number of replacements over time, by diagnosis group.

The annual number of total wrist replacements changed over time (Figure 2). A decrease in the number of arthroplasties due to inflammatory arthritis was found (p < 0.001), but operations due to noninflammatory arthritis increased (p < 0.001).

### Type of prosthesis

The Biax prosthesis was used almost exclusively in patients in the I group (Table 1). The median follow-up time was longer for the Biax prosthesis (9.3 years) than for the Elos (4.6 years) and Gibbon (2.6 years) (p < 0.001). The Elos prosthesis was used exclusively in the NI group, and the Gibbon prosthesis was used in both groups.

### Revision and survival

39 (21%) of the 189 wrist prostheses were revised: 10 of 23 Elos prostheses, 11 of 76 Gibbon prostheses, and 18 of 90 Biax prostheses.

**Figure 3.**
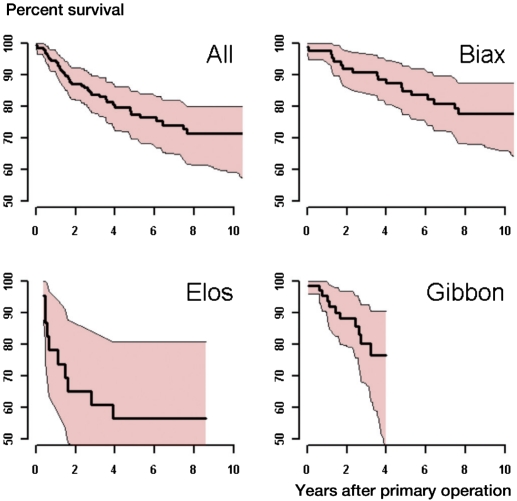
Total and prosthesis-specific survival (Kaplan-Meier) with 95% CI shown in red.

The mean time until first revision was 9.1 (CI: 8.5–9.8) years. The overall 5-year and 10-year survival was 78% (CI: 70–85) and 71% (CI: 59–80), respectively (Figure 3). Prosthesis survival was 85% (CI: 78–93) at 5 years for the Biax prosthesis, 77% (CI: 30–90) at 4 years for the Gibbon prosthesis, and 57% (CI: 33–81) at 5 years for the Elos prosthesis (Figure 3).

Loosening of the distal component and pain were the most common reasons for revision (Table 2). For the Biax prosthesis, the main causes of revision were aseptic loosening, incorrect axis, and pain. The main cause of revision of the Elos and Gibbon prostheses was aseptic loosening of the distal component, but in 3 cases deep infection was the reason for revision of the Gibbon prosthesis. In no case was pain registered as the only cause of revision.

**Table 2.** Reasons for revision (more than one reason could be given)

Brand	Biax	Elos	Gibbon	Total
Loosening of proximal component	3	2		5
Loosening of distal component	8	8	5	21
Dislocation	2	—	—	2
Instability	3	—	—	3
Axis problems	7	—	1	8
Deep infection	1	—	3	4
Pain	7	1	2	10
Wear of liner	1	—	—	1
Total number of revisions	18	10	11	39

In the 39 patients who underwent revision, the procedures performed were: exchange of the distal component (n = 9), exchange of the proximal component (n = 3), exchange of the whole prosthesis (n = 2), and removal of prosthetic parts without replacement (n = 23). An unknown revision procedure was done in 2 wrists.

### Risk factors for revision

Females had higher revision rate than males (RR = 3, CI: 1–7). Age had no statistically significant influence on prosthesis survival (RR = 0.8 per 10 year, CI: 0.66–1.1). No statistically significant difference in survival was found for the patients in the two diagnosis groups (RR = 1.2, CI: 0

Only 7 hospitals in Norway performed wrist arthroplasty surgery. The number of procedures performed in each hospital during the observation period ranged from one to 70 prostheses. The revision rate was similar in hospitals that had performed more than or less than 50 wrist arthroplasties.

## Discussion

The 5- and 10-year survival rates of 78% and 71% are similar to those in other studies. In a study of 76 wrist replacements with the Biax prosthesis, the 8-year survival of the implant was found to be 83% (Takwale et al. 2002). The 5-year survival was also 83% in a study of 52 patients treated with the Biax prosthesis (Cobb and Beckenbaugh 1996). The patients in these 3 studies were also similar to ours regarding age, sex, and diagnoses.

### Completeness of data

The completeness of registration in the Norwegian Arthroplasty Register was recently evaluated by comparing it to the mandatory reporting of administrative data to the Norwegian Patient Register (NPR), and it was found to be 97% for hip replacements, 99% for knee replacements, 82% for all primary ankle replacements, but only 52% for wrist replacements. One explanation for the under-reporting could be that relative to the hip and knee, few wrist replacements are performed, and that for this reason reporting to the Arthroplasty Register is not so well established among wrist surgeons.

The magnitude of under-reporting is unclear, however, as the NBD code group (except for NBD 8) of the NOMESCO 2006 coding system, which is used by the hospitals in their reports to the NPR, does not require it to be specified whether the prosthesis has been inserted in the radio-carpal joint or in other joints in the carpus. Also, the code NBD 99 applies to any prosthesis operation in the wrist or hand. Thus, the NPR data on wrist implants most probably also include data from implants in joints other than the radio-carpal joint (Espehaug et al. 2006). We have no reason to believe that there is any systematic under-reporting to the NAR.

### Time trends

The overall incidence of reported wrist arthroplasties was unchanged in Norway during the study period (Figure 3). The incidence of total wrist replacements increased with time in the NI group, but not in the I group. These findings are consistent with a general trend in recent years, which has also been seen for other joints. More joint replacements are being performed due to osteoarthritis, and less for inflammatory arthritis (da Silva et al. 2003, Pedersen et al. 2005, Weiss et al. 2006, Fevang et al. 2007).

### Risk factors for revision

The only factor that statistically significantly influenced survival was sex: females had a 3-fold higher revision rate than males. We found that there was no difference in prosthesis survival in patients with different diagnoses, which is in accordance with the results of previous studies on ankle prostheses (Spirt et al. 2004, Doets et al. 2006).

### Prosthesis type and survival

There were major differences between the prosthesis types concerning patient demographics and inclusion periods. In the Elos group, three different prostheses were used in the 23 patients; the numbers were too small to allow us to perform a meaningful comparison of the different versions. In the Elos group, only non-inflammatory wrists were included, and in the Biax group only inflammatory wrists were included. In the Gibbon group, there was a mixture of the two diagnostic groups. In addition, the number of patients with inflammatory joint disease in the Gibbon group was small. Furthermore, the 3 versions of the Elos prosthesis were all preliminary types of the Gibbon prosthesis. For these reasons, and due to the small numbers, our results on differences between types should be interpreted with caution. Based on our findings, we cannot conclude that one type of prosthesis was superior to any another. The results of wrist arthroplasty are still inferior to those of knee and hip arthroplasty, and the function may not be substantially better than with arthrodesis. Current evidence does not support widespread implementation of this procedure.

## References

[CIT0001] Brase DW, Millender LH (1986). Failure of silicone rubber wrist arthroplasty in rheumatoid arthritis. J Hand Surg Am.

[CIT0002] Carlson JR, Simmons BP (1998). Wrist arthrodesis after failed wrist implant arthroplasty. J Hand Surg Am.

[CIT0003] Cavaliere CM, Chung KC (2008). A systematic review of total wrist arthroplasty compared with total wrist arthrodesis for rheumatoid arthritis. Plast Reconstr Surg.

[CIT0004] Cobb TK, Beckenbaugh RD (1996). Biaxial total-wrist arthroplasty. J Hand Surg Am.

[CIT0005] da Silva E, Doran MF, Crowson CS, O'Fallon WM, Matteson EL (2003). Declining use of orthopedic surgery in patients with rheumatoid arthritis? Results of a long-term, population-based assessment. Arthritis Rheum.

[CIT0006] Doets HC, Brand R, Nelissen RG (2006). Total ankle arthroplasty in inflammatory joint disease with use of two mobile-bearing designs. J Bone Joint Surg (Am).

[CIT0007] Dorey FJ, Korn EL (1987). Effective sample sizes for confidence intervals for survival probabilities.Stat Med.

[CIT0008] Espehaug B, Furnes O, Havelin LI, Engesaeter LB, Vollset SE, Kindseth O (2006). Registration completeness in the Norwegian Arthroplasty Register. Acta Orthop.

[CIT0009] Fevang BT, Lie SA, Havelin LI, Engesaeter LB, Furnes O (2007). Reduction in orthopedic surgery among patients with chronic inflammatory joint disease in Norway, 1994-2004. Arthritis Rheum.

[CIT0010] Havelin LI (1999). The Norwegian Joint Registry. Bull Hosp Jt Dis.

[CIT0011] Jolly SL, Ferlic DC, Clayton ML, Dennis DA, Stringer EA (1992). Swanson silicone arthroplasty of the wrist in rheumatoid arthritis: a long-term follow-up. J Hand Surg Am.

[CIT0012] Lorei MP, Figgie MP, Ranawat CS, Inglis AE (1997). Failed total wrist arthroplasty. Analysis of failures and results of operative management. Clin Orthop.

[CIT0013] Meuli HC (1973). Arthroplasty of the wrist. Ann Chir.

[CIT0014] Pedersen AB, Johnsen SP, Overgaard S, Soballe K, Sorensen HT, Lucht U (2005). Total hip arthroplasty in Denmark: incidence of primary operations and revisions during 1996-2002 and estimated future demands. Acta Orthop.

[CIT0015] Schemper M, Smith TL (1996). A note on quantifying follow-up in studies of failure time. Control Clin Trials.

[CIT0016] Smith RJ, Atkinson RE, Jupiter JB (1985). Silicone synovitis of the wrist. J Hand Surg Am.

[CIT0017] Spirt AA, Assal M, Hansen ST (2004). Jr. Complications and failure after total ankle arthroplasty. J Bone Joint Surg (Am).

[CIT0018] Swanson AB (1973). Flexible implant arthroplasty for arthritic disabilities of the radiocarpal joint. A silicone rubber intramedullary stemmed flexible hinge implant for the wrist joint. Orthop Clin North Am.

[CIT0019] Takwale VJ, Nuttall D, Trail IA, Stanley JK (2002). Biaxial total wrist replacement in patients with rheumatoid arthritis. Clinical review, survivorship and radiological analysis. J Bone Joint Surg (Br).

[CIT0020] Vogelin E, Nagy L (2003). Fate of failed Meuli total wrist arthroplasty. J Hand Surg Br.

[CIT0021] Volz RG (1976). The development of a total wrist arthroplasty. Clin Orthop.

[CIT0022] Weiss RJ, Stark A, Wick MC, Ehlin A, Palmblad K, Wretenberg P (2006). Orthopaedic surgery of the lower limbs in 49,802 rheumatoid arthritis patients: results from the Swedish National Inpatient Registry during 1987 to 2001. Ann Rheum Dis.

